# Mutation of a Cuticle Protein Gene, *BmCPG10*, Is Responsible for Silkworm Non-Moulting in the 2^nd^ Instar Mutant

**DOI:** 10.1371/journal.pone.0153549

**Published:** 2016-04-20

**Authors:** Fan Wu, Pingyang Wang, Qiaoling Zhao, Lequn Kang, Dingguo Xia, Zhiyong Qiu, Shunming Tang, Muwang Li, Xingjia Shen, Guozheng Zhang

**Affiliations:** 1 School of Biotechnology, Jiangsu University of Science and Technology, Zhenjiang, 212018, China; 2 The Sericulture Research Institute, Chinese Academy of Agricultural Sciences, Zhenjiang, 212018, China; 3 Industrial Crops Institute, Hubei Academy of Agricultural Sciences, Wuhan, 430064, China; Institute of Plant Physiology and Ecology, CHINA

## Abstract

In the silkworm, metamorphosis and moulting are regulated by ecdysone hormone and juvenile hormone. The subject in the present study is a silkworm mutant that does not moult in the 2^nd^ instar (*nm2*). Genetic analysis indicated that the *nm2* mutation is controlled by a recessive gene and is homozygous lethal. Based on positional cloning, *nm2* was located in a region approximately 275 kb on the 5^th^ linkage group by eleven SSR polymorphism markers. In this specific range, according to the transcriptional expression of thirteen genes and cloning, the relative expression level of the *BmCPG10* gene that encodes a cuticle protein was lower than the expression level of the wild-type gene. Moreover, this gene’s structure differs from that of the wild-type gene: there is a deletion of 217 bp in its open reading frame, which resulted in a change in the protein it encoded. The *BmCPG10* mRNA was detectable throughout silkworm development from the egg to the moth. This mRNA was low in the pre-moulting and moulting stages of each instar but was high in the gluttonous stage and in newly exuviated larvae. The *BmCPG10* mRNA showed high expression levels in the epidermis, head and trachea, while the expression levels were low in the midgut, Malpighian tubule, prothoracic gland, haemolymph and ventral nerve cord. The ecdysone titre was determined by ELISA, and the results demonstrated that the ecdysone titre of *nm2* larvae was lower than that of the wild-type larvae. The *nm2* mutant could be rescued by feeding 20-hydroxyecdysone, cholesterol and 7—dehydrocholesterol (7dC), but the rescued *nm2* only developed to the 4^th^ instar and subsequently died. The moulting time of silkworms could be delayed by *BmCPG10* RNAi. Thus, we speculated that the mutation of *BmCPG10* was responsible for the silkworm mutant that did not moult in the 2^nd^ instar.

## Introduction

Metamorphosis and moulting is a phenomenon particular to insects, the growth and development of which includes periodic moulting. The moulting is regulated by many hormones, of which the most important are the ecdysone hormone and juvenile hormone. The exoskeleton is rebuilt by the degradation of old cuticle protein followed by the synthesis of new cuticle protein during insect moulting, and the signalling pathways of moulting hormone play an important role in this process [1–3]. Therefore, the cuticle protein genes are critical target genes of ecdysone, and their expression is closely related to the moulting and metamorphosis of insects. Recent studies have shown that the 1^st^ and 2^nd^ instars of silkworm are JH-independent phases in which JH does not have an important function [4].

Insect moulting involves the orderly expression of a series of genes, and the abnormalexpression of these genes might result in non-moulting. Several non-moulting silkworm mutants have been discovered [5, 6], including non-moulting *nm* (11–13.8) [7], non-moulting dwarf *nm-d* (9–16.3) [8], and non-moulting Matsuno *nm-m*(10–27.9)[9]. The mutation gene responsible for the mutant known as non-moulting glossy *nm-g* (17–39.1) [10] silkworm *Bombyx mori* has been studied, and *nm-g* was identified as the key gene responsible for the mutant. This gene encodes a short chain dehydrogenase and takes part in the ‘Black Box’ for the synthesis of ecdysone hormone [11]. Most of these mutants were non-moulting in the 1^st^ instar. The silkworm mutant albino (al) is a lethal mutant with a colourless cuticle after the first ecdysis and dies without feeding on mulberry [12]. Sora Enya et al. used TALEN-mediated genome editing to generate a *B*. *mori* genetic mutant of *nobo-Bm*, which results in a 2^nd^ instar with a glossy cuticle that cannot undergo moulting and cannot develop into the 3^rd^ instar [13]. In addition, a mutation in *GSTe7* results in the accumulation of 7-dehydrocholesterol.

We discovered a new mutant of non-moulting in the 2^nd^ instar (*nm2*) from the silkworm variety C603. The mutant develops normally in the 1^st^ instar and moults on time, but in the beginning of the pre-moulting stage of the 2^nd^ instar, the mutant larvae become lustrous, last for 6–8 days with hardly any development, cannot moult and exuviate and finally die. Genetic analysis revealed that *nm2* was controlled by a single recessive gene [14]. In the present study, we mapped the candidate gene involved in the *nm2* mutant by positional cloning and identified *BmCPG10* as the gene most likely responsible for the *nm2* mutant, *BmCPG10* had a transcriptional expression level in *nm2* that was lower than that of the wild-type gene. The *BmCPG10* ORF of *nm2* had a 217 bp deletion compared with the wild-type gene; thus, the structure of *BmCPG10* and the protein encoded by *BmCPG10* were altered. The titre of ecdysone in *nm2* was lower than that of the wild-type, which resulted in the inability to moult in the 2^nd^ instar. These results might provoke further investigations into the molecular mechanisms of insect moulting and increase the understanding of the role of cuticle proteins in the moulting process of insects.

## Materials and Methods

### Silkworm strains

Silkworm strain p50 (standard silkworm strain), the wild-type C603 and *nm2* mutant strain, were supplied by the Sericultural Research Institute (Zhenjiang, China). The larvae were reared on fresh mulberry leaves at (25 ±2)°C under a 12-h light/12-h dark photoperiod and 65 ±5% relative humidity.

### Establishment of positional cloning group

The female parent (P_1_) was selected from an inbred line of p50, and the male parent (P_2_) with the genotype of *+/nm2* was selected because of the lethality of *nm2/nm2*. A single-pair cross between p50 and P2 produced the F_1_ offspring, and a positive and negative backcross (BC_1_) between F_1_ and P_2_ produced the BC_1_F and BC_1_M. Eleven normal and ten mutant BC_1_F progenies from the same parents were used for the linkage analysis. The 594 mutants of BC_1_M were used for the recombination analysis.

### Genomic DNA extraction

Genomic DNA of the parents (P_1_ and P_2_), F_1_ individuals, and BC_1_F normal individuals were isolated from the silk gland, while the individual mutants of BC_1_F and BC_1_M were isolated from the whole 2^nd^ larvae without the midgut. DNA was extracted according to previously described methods [15]. The quality of the genomic DNA was determined at a 260/280 absorbance ratio, and the concentration of the genomic DNA was diluted to 100 ng/μL and stored at −20°C.

### Linkage and recombination analysis

Simple sequence repeat (SSR) markers were obtained from the published SSR linkage map [16]. Polymorphisms of these SSR markers were detected by agarose gel electrophoresis. Eleven normal and ten mutant BC_1_F progenies from the same parents were used for the linkage analysis and preliminary SSR mapping. If the banding patterns of eleven normal BC_1_F progenies were inaccordance with the banding patterns of F_1_ or P_2_ without P1, and if the banding patterns of ten mutant BC_1_F progenies were inaccordance with the banding patterns of P_2_, then a linkage between *nm2* and the SSR marker was indicated.

We downloaded the up- and down-stream sequences (http://sgp.dna.affrc.go.jp/) close to the tightly linked SSR marker of the *nm2* locus and searched for new SSR markers using SSR Hunter 1.3 based on the results of the preliminary SSR mapping. Five hundred ninety four mutant BC_1_M individuals and new markers that exhibited polymorphism among the parents and F_1_ individuals were used for fine mapping. The primers for the SSR markers with polymorphism are listed in S1 and S2 Tables.

### Semi-quantitative and quantitative real-time PCR

Total RNAs were extracted from the pre-moulting silkworm of 2^nd^ instar of the wild-type and *nm2* mutants, the larvaes at different developmental stages from eggs to moth, and various tissues including epidermis, fat body, midgut, silk gland, malpighian tubule, trachea, head, hemocytes, testis, ovary, muscle, prothoracic gland and ventral nerve cord from the third day in the fifth instar larvae of wild-type using RNAiso Plus (TaKaRa). The quality of the total RNAs was determined at a 260/280 absorbance ratio, as well as with the electrophoresis method, after which the RNA was stored at −80°C. After treatment with DNaseI, 1 μg of the total RNA was used to synthesize the first strand cDNA using a Primerscript Reverse Transcriptase kit (TaKaRa) according to the manufacturer's protocol. The primers of the 13 candidate genes for semi-quantitative reverse-transcription polymerase chain reaction (RT-PCR) were listed in S3 Table. The silkworm housekeeping gene *Bm-actin A3* was used as an internal control for normalization of sample loading.

qRT-PCR was performed using an ABI PRISM^®^ 7300 Sequence Detection System (Applied Biosystems, USA). *BmCPG10* mRNA and *Bm-actin A3* mRNA were quantified using 2 μL of the reverse transcription reaction (equivalent to 100 ng single stranded cDNA) as a template in qRT-PCR. A 289 bp product for *BmCPG10* cDNA was amplified using the following primers: forward 5-GGCGTCTATTGGTGATGGTGATAAC -3 and reverse 5-GAGTCCAAAGAACAAGGTTCGCTTC -3. qRT-PCR was performed in a 20 μL reaction mixure using SYBR Green Supermix (Takara), according to the manufacturer’s instructions. The thermal cycling profile consisted of initial denaturation at 94°C for 5 min; and 40 cycles at 94°C for 30 s, 60°C for 25 s, and 72°C for 35 s. All of the reactions were performed in triplicate, and the relative expression levels were analysed using the 2^−ΔΔCt^ method, where ΔΔCt = ΔCt _sample_− ΔC _treference_, and Ct refers to the cycle threshold [17]. The silkworm housekeeping gene *Bm-actin A3* was used as an internal control for the normalization of sample loading.

### RNAi reduction *BmCPG10* mRNA experiment

The 373 bp fragment of *BmCPG10* cDNA was amplified using the wild-type silkworm cDNA as a template for RNA synthesis with the following primers containing the promoter of T7: forward: 5-TAATACGACTCACTATAGGGAAGGGACATCTTGAACC-3, reverse: 5-TAATACGACTCACTATAGGCGCAAAGTCACAGGAAAC-3. The dsRNA was synthesized using a MEGA script RNAi kit (Ambion). RNA synthesis and purification were performed according to the manufacturer’s instructions, and the integrity of dsRNA was confirmed by nondenaturing agarose gel electrophoresis. The quality of the dsRNA was determined at a 260/280 absorbance ratio, and the dsRNA was diluted to a final concentration of 2000 ng/μL with an injection buffer. *EGFP* dsRNA was synthesized using the method of Quan et al [18].

### Annotation analysis

The candidate genes in the region narrowed by the linkage analysis were annotated using the silkworm genome database(http://sgp.dna.affrc.go.jp/KAIKObase) and BLASTX from the National Center for Biotechnology Information (http://blast.ncbi.nlm.nih.gov/Blast.cgi).

### Assay of ecdysteroid titres

The silkworms that were collected above were frozen and homogenized in 50% MeOH (600μL). The homogenate was centrifuged, and the supernatant was stored at -20°C. Ecdysteroid titres were assayed using an Insect Ecdysone ELISA Kit (Shanghai Meilian Bio Technology Co., Ltd.) according to the manufacturer’s instructions. The quality of the protein was determined at a 280 absorbance ratio, and the concentration of the protein was diluted to 1 mg/mL and stored at −20°C.

### Feeding experiments

After the separation of the non-moulting larvae during 2^nd^ instar, we fed mulberry leaves supplemented with 400 mg/L 20-hydroxyecdysone (20E), 8000 mg/L cholesterol and 7—dehydrocholesterol (7dC) to *nm2*. Supplementation with water was used as a control. The specific concentrations of 20E, cholesterol and 7dC were dependent on the preliminary feeding experiment that listed in the S4 Table. For each group, including 50 *nm2* mutants, the experiment was repeated at least three times.

## Results

### The *nm2* gene was located on the 5^th^ linkage group

As chromatid exchanges do not occur in female silkworms and the *nm2* mutation is lethal, we used the *+/nm2* instead of *nm2/nm2* as P_2_ to establish the positional cloning group. The genotype of F_1_ generation was +/*nm2* and +/*+*, so there was no mutant separated from the F_1_ generation. There was no mutant separated from the group of BC_1_F and BC_1_M when the genotype of F_1_ generation was +/*+*, so this group of BC_1_F and BC_1_M were not used for positional cloning. And the BC_1_F and BC_1_M that had mutant separated from were used for positional cloning. Polymorphic markers on each linkage group of silkworm were found (listed in S1 Table), These polymorphic markers as well as 11 normal and 10 *nm2* BC_1_F progenies that from the same parents were used to determine the linkage group on which the *nm2* gene was located. According to Mendel genetic law, if a polymorphic marker was linked with the *nm2* gene, then the banding patterns of 11 normal BC_1_F progenies were consistent with the F_1_ or P_2_ individuals, and the banding patterns of 10 *nm2* BC_1_F progenies from the same parents were consistent with the P_2_ individuals.

**Fig 1:**
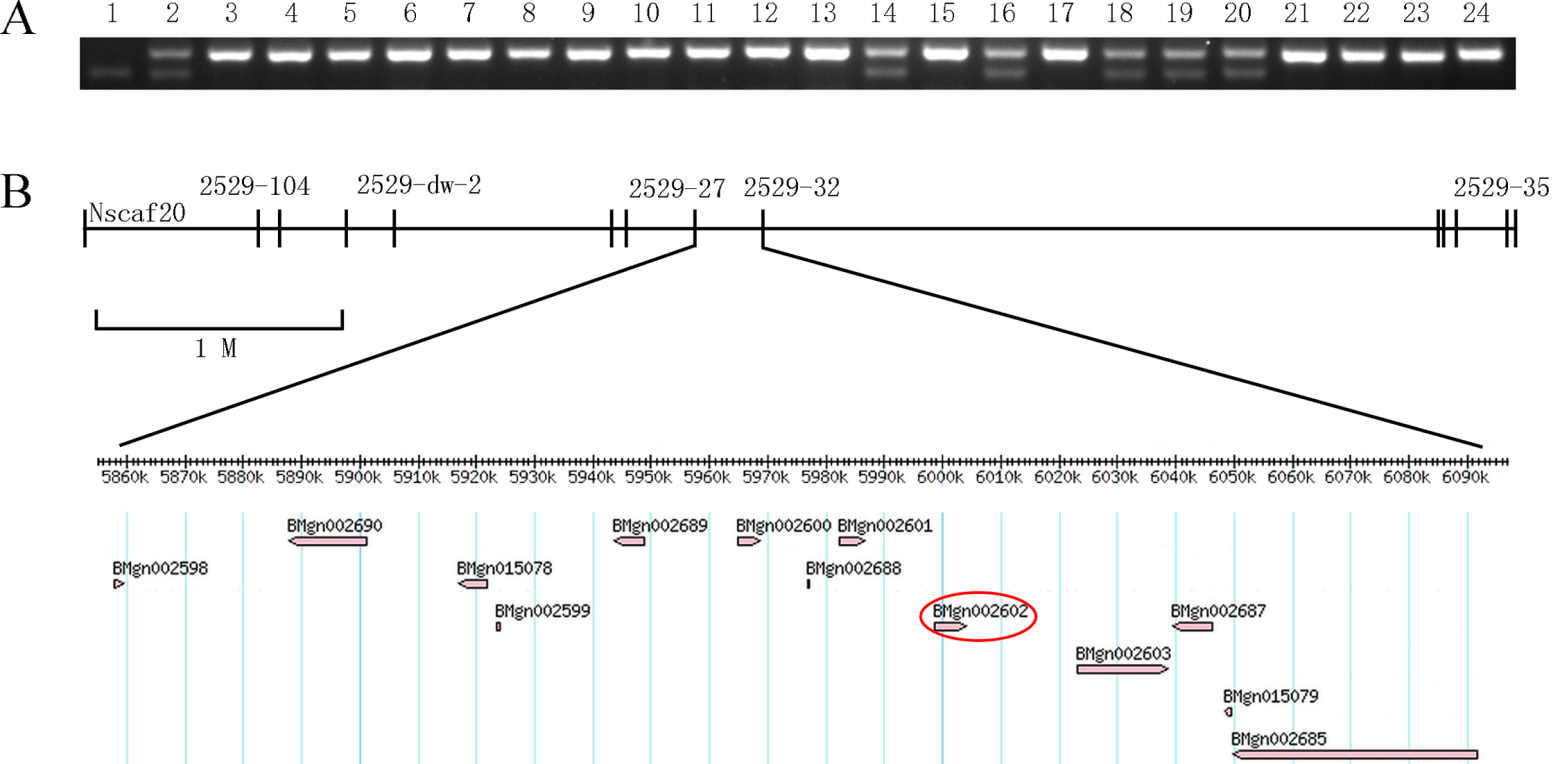
The genetic linkage map of *nm2*. (A) Inheritance pattern of *nm2* gene linked SSR marker S2529-2 in BC_1_F progeny. Lane 1: p50 parent (P_1_), lane 2: F_1_ (p50×*nm2*), lane 3: *nm2* mutant parent (P_2_), lanes 4–13: 10 *nm2* mutants of BC_1_F progenies, lanes 14–24: 11 wild-type of BC_1_F progenies. (B) Physical map showing the outcome of the linkage analysis using 594 BC_1_M individuals. The *nm2* locus was narrowed to the genomic region flanked by the SSR markers S2529-32 and S2529-27. Putative genes are shown below the map, and *BmCPG10 (BMgn002602)* is shown in a red circle.

The results of marker S2529-2 indicated that 11 normal BC_1_F progenies exhibited a banding pattern similar to that of F_1_ or P_2_, whereas 10 *nm2* BC_1_F progenies exhibited a banding pattern similar to that of P_2_ (Fig 1A). The results of other polymorphic markers that we found and located on the other linkage group indicated that11 normal and 10 *nm2* BC_1_F progenies all exhibited a banding pattern similar to that of F_1_ or P_2_. Thus, the *nm2* gene was located on the 5^th^ linkage group, and the marker S2529-2 was linked to the *nm2* gene. Based on the linkage analysis, eleven SSR markers were developed on the 5^th^ chromosome, including S2529-43, S2529-45, S2529-46, S2529-32, S2529-35, S2529-27, S2529-91, S2529-2, S2529-121, S2529-74 and S2529–104 (S2 Table), and 594 *nm2* BC_1_M individuals were used for fine mapping. The results indicated that *nm2* was located in a region of approximately 275 kb on the *Bm*_ nscaf20 from 2496529 bp to 2772188 bp compared with the silkworm genome database KAIKObase. Thirteen genes within the particular region were predicted using gene-prediction models, including *BMgn002598*, *BMgn002690*, *BMgn015078*, *BMgn002599*, *BMgn002689*, *BMgn002600*, *BMgn002688*, *BMgn002601*, *BMgn002602*, *BMgn002603*, *BMgn002687*, *BMgn015079*, and *BMgn002685* (Fig 1B).

### *BmCPG10* is the *nm2* key gene

**Fig 2:**
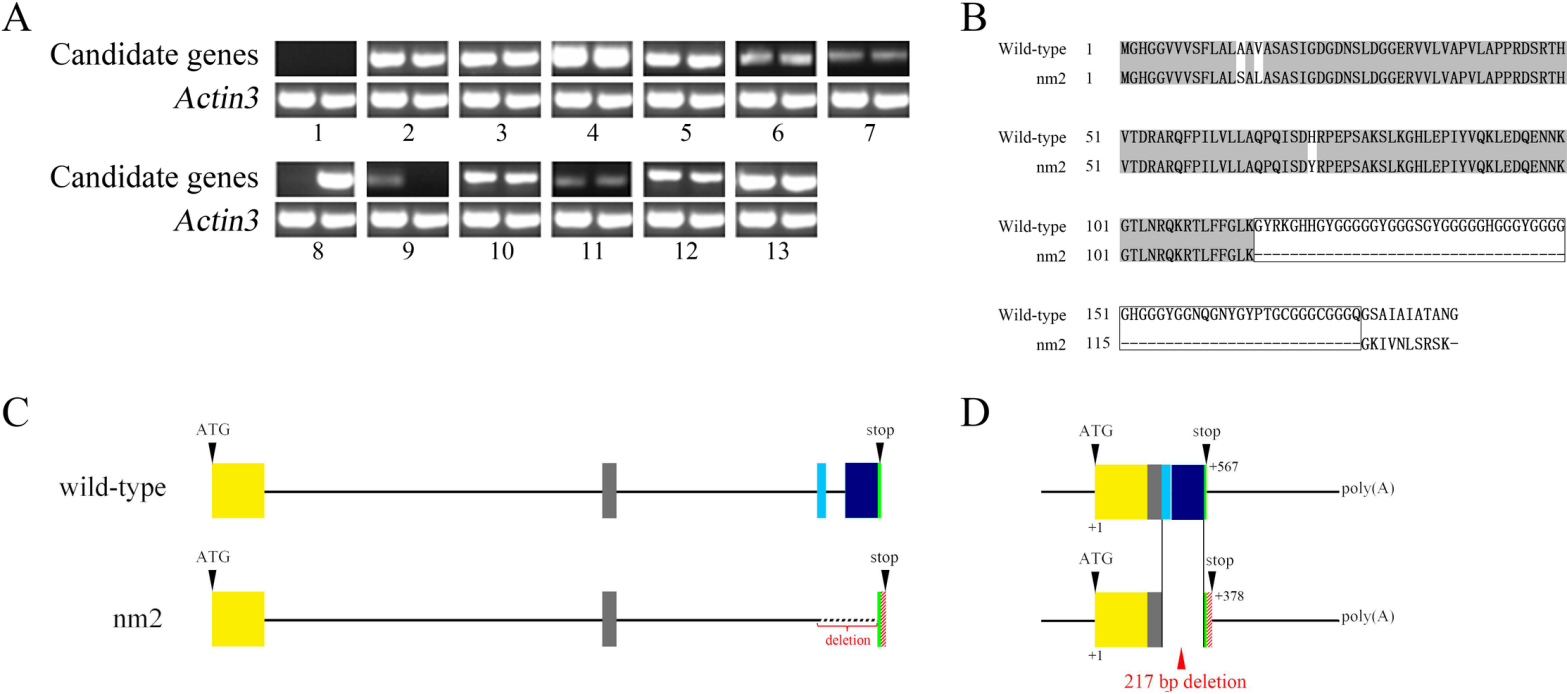
The expression and amino acid sequence of the *nm2* candidate gene. (A) Expression profiles of *nm2* candidate genes based on semi-quantitative RT-PCR. 1–13 representing 13 genes: *BMgn002598*, *BMgn002690*, *BMgn015078*, *BMgn002599*, *BMgn002689*, *BMgn002600*, *BMgn002688*, *BMgn002601*, *BmCPG10 (BMgn002602)*, *BMgn002603*, *BMgn002687*, *BMgn015079* and *BGIBMGA002685*. The *Bm-actin A3* gene was used as an internal control. Left lane of each map is wild-type and right lane of each map is *nm2* mutant. (B) Amino acid sequence alignments for wild-type and *nm2*. The boxes indicate the amino acid deletions in *nm2*. (C) The top gene structure is the wild-type and the bottom structure is the *nm2* mutant. The boxes indicate exons for the coding region and the line for the noncoding region. Start and stop codons are indicated by ATG and stop. The deletion of 217 bp in *nm2* is indicate by a red dotted line. (D) The top is the full -length cDNA of wild-type, and the bottom is the full -length cDNA of *nm2*.

Because *nm2* was non-moulting in the 2^nd^ instar, we analysed the expression profiles of the thirteen initial candidate genes of 2^nd^ instar pre-moulting silkworm between *nm2* and the wild-type by semi-quantitative RT-PCR (Fig 2A). Only the gene *BMgn002598* was not expressed in both *nm2* and wild-type. The gene *BMgn002601* was not expressed in wild-type but was expressed in *nm2*, while the gene *BMgn002602* was not expressed in *nm2* but was expressed in wild-type. There were no differences in the expression of the other genes.

We obtained the open reading frame (ORF) of *BMgn002601* and *BMgn002602* from the *nm2* mutant and wild-type strains using primers designed for the ORF of *BMgn002601* and *BMgn002602*. We found that the *BMgn002601* ORF of *nm2* and of the wild-type was identical, but the *BMgn002602* ORF in *nm2* involved a deletion of 217 bp and resulted in a difference in the amino acid sequence of *nm2* compared with the wild-type (Fig 2). *BMgn002601* encodes a hypothetical protein 35 with a function that is unclear. *BMgn002602* encodes a cuticle protein, BmCPG1*0* [19], rich in glycine according to the silkworm database (http://sgp.dna.affrc.go.jp/KAIKObase) and GenBank of NCBI(http://blast.ncbi.nlm.nih.gov/Blast.cgi). The 217 bp deletion of *BMgn002602* was located in the region that rich in glycine, which might destroy the functional domain of the *BMgn002602*. The deletion resulted in the backward of the termination codon, and the mutated gene was predicted to encode a new protein.

**Fig 3:**
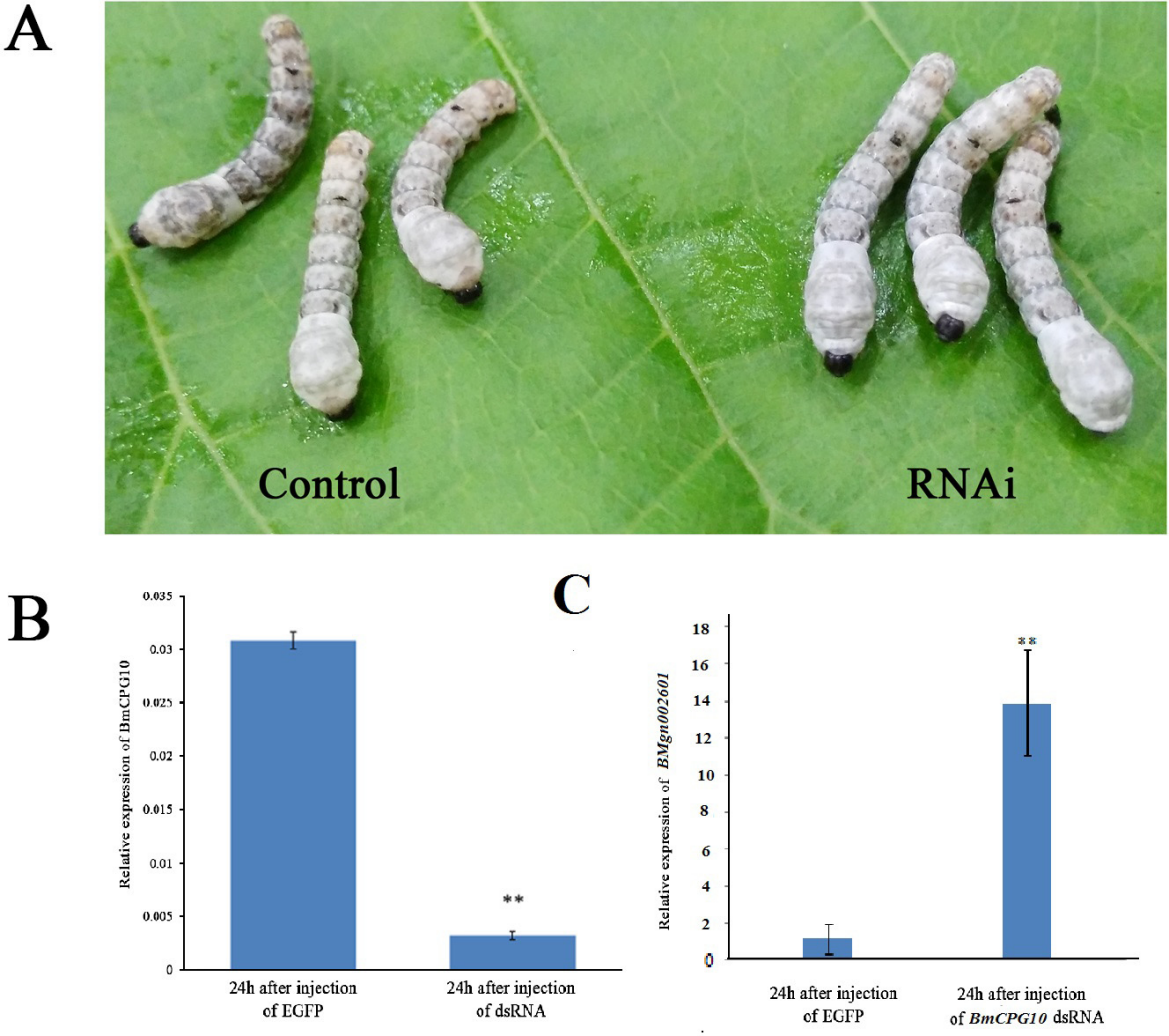
The results of *BmCPG10* RNAi. (A) The developmental condition of silkworm 24 h after injection. The left was injected with EGFP and was moulting. The right was injected with dsRNA of *BmCPG10* and was pre-moulting. (B) Relative expression levels of *BmCPG10* 24 h after injection, as determined by quantitative real-time PCR. Each real-time PCR analysis was repeated at least three times for each set of RNA samples. (C) Relative expression levels of *BMgn002601* 24 h after injection of *BmCPG10* and *EGFP* dsRNA, as determined by quantitative real-time PCR. Each real-time PCR analysis was repeated at least three times for each set of RNA samples. Each point represents the mean value ±SD. The relative amounts of *BmCPG10* were determined using the *Bm-actin A3* as a standard. **indicates significant difference (p < 0.01) compared with the control.

To determine whether *nm2* was caused by decreasing the amount of *BmCPG10* mRNA, we synthesized dsRNA corresponding to a portion of the *BmCPG10* ORF and injected it into the wild-type larvae during the gluttonous stage of 2^nd^ instar at doses of 2000 ng per individual; we injected the same doses of *EGFP* dsRNA as a control. Four of the 10 individuals injected with *BmCPG10* dsRNA were delayed in their moulting time during the 2^nd^ instar, while the controls moulted24 h after the injection (Fig 3A). The moulting time of 4 individuals injected with *BmCPG10* dsRNA in the 2^nd^ instar could be delayed 48 h to 72 h. Using qRT-PCR, we compared the expression of *BmCPG10* and *BMgn002601* mRNA among individuals injected with *BmCPG10* and *EGFP* dsRNA. The expression level of *BmCPG10* mRNA in the 4 individuals injected with *BmCPG10* dsRNA was significantly lower than that of the controls (Fig 3B) and the expression level of *BMgn002601* mRNA in the 4 individuals injected with *BmCPG10* dsRNA was significantly higher than that of the controls (Fig 3C). These results showed that the expression level of *BMgn002601* mRNA could be influenced by the decreasing amount of *BmCPG10* mRNA. Thus the expression of *BMgn002601* mRNA in *nm2* might be caused by the mutantion of *BmCPG10*.

We therefore concluded that the *BmCPG10* gene was the most likely candidate for the *nm2* mutant.

### The developmental and tissue-specific transcription pattern of *BmCPG10*

**Fig 4:**
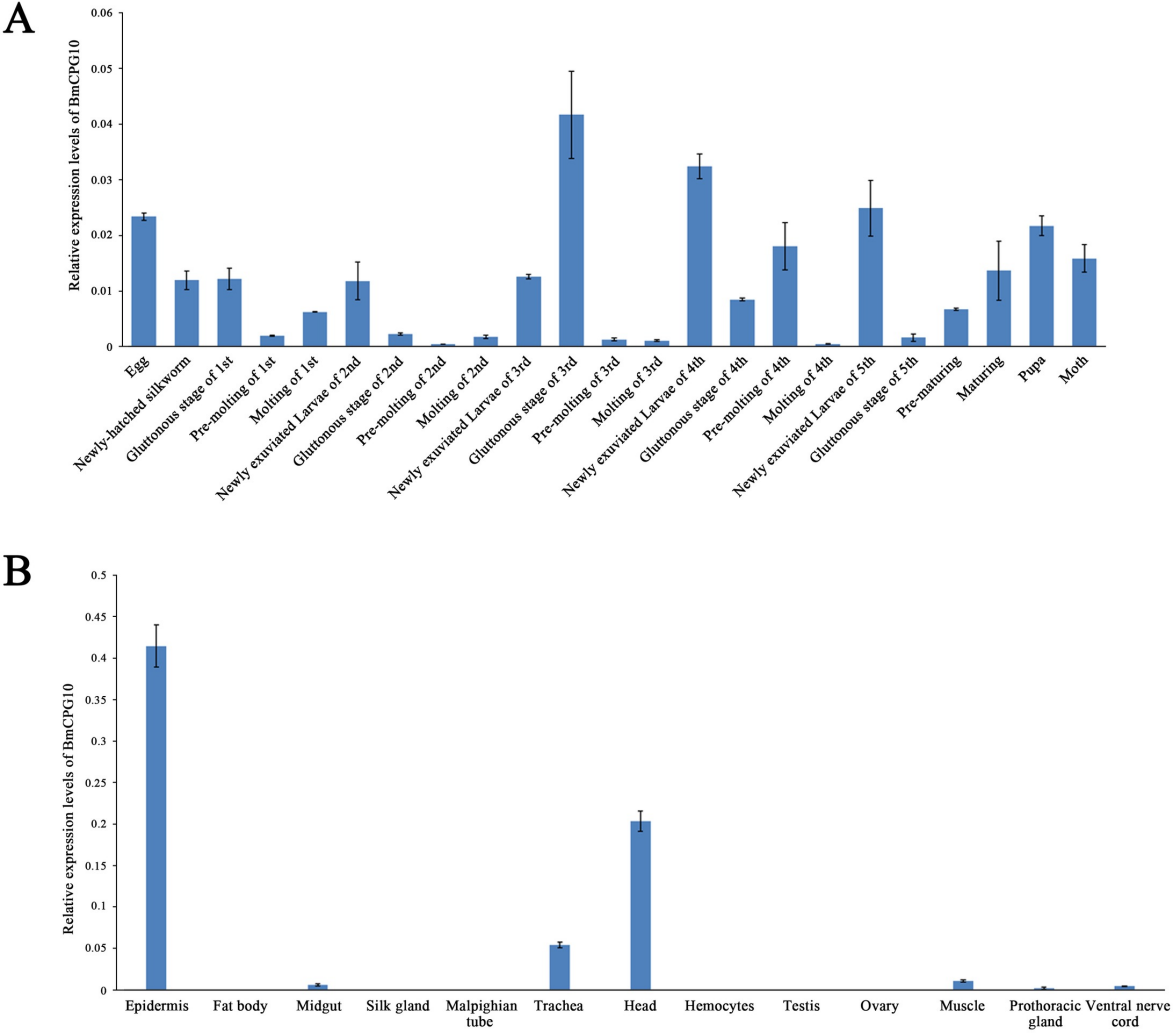
The relative expression levels of *BmCPG10*, as determined by quantitative real-time PCR. (A) The relative expression levels of *BmCPG10* at various developmental stages of wild-type. (B) The relative expression levels of *BmCPG10* indifferent tissues in the wild-type larvae. Each real-time PCR analysis was repeated at least three times for each set of RNA samples. Each point represents the mean value ±SD. The relative amounts of *BmCPG10* were determined using the *Bm-actin A3* as a standard.

The expression profiles of *BmCPG10* at various developmental stages of wild-type were examined using the entire body, except for the midgut of the larvae by qRT-PCR. The results indicated that *BmCPG10* mRNA was detectable throughout the development, from the egg to the moth. The *BmCPG10* mRNA expression was low during the pre-moulting and moulting of each instar, and was highest during the gluttonous stage of 3^rd^ instar and was lowest during the pre-moulting of 2^nd^ instar (Fig 4A). To determine the tissue specificity of *BmCPG10* mRNA expression in the larvae, total RNA from the third day of the fifth instar larvae of C603 was isolated from the epidermis, fat body, midgut, silk gland, Malpighian tubule, trachea, head, haemocytes, testis, ovary, muscle, prothoracic gland and ventral nerve cord and then was subjected to qRT-PCR. The results showed that the *BmCPG10* gene transcription could be detected in most of the tissues. The epidermis, head and trachea had higher levels, while the midgut, Malpighian tubule, prothoracic gland and ventral nerve cord had lower levels, however the *BmCPG10* gene transcript was barely detected in the haemocytes (Fig 4B).

### The low titre of ecdysone in *nm2* resulted in the mutant phenotype

**Fig 5:**
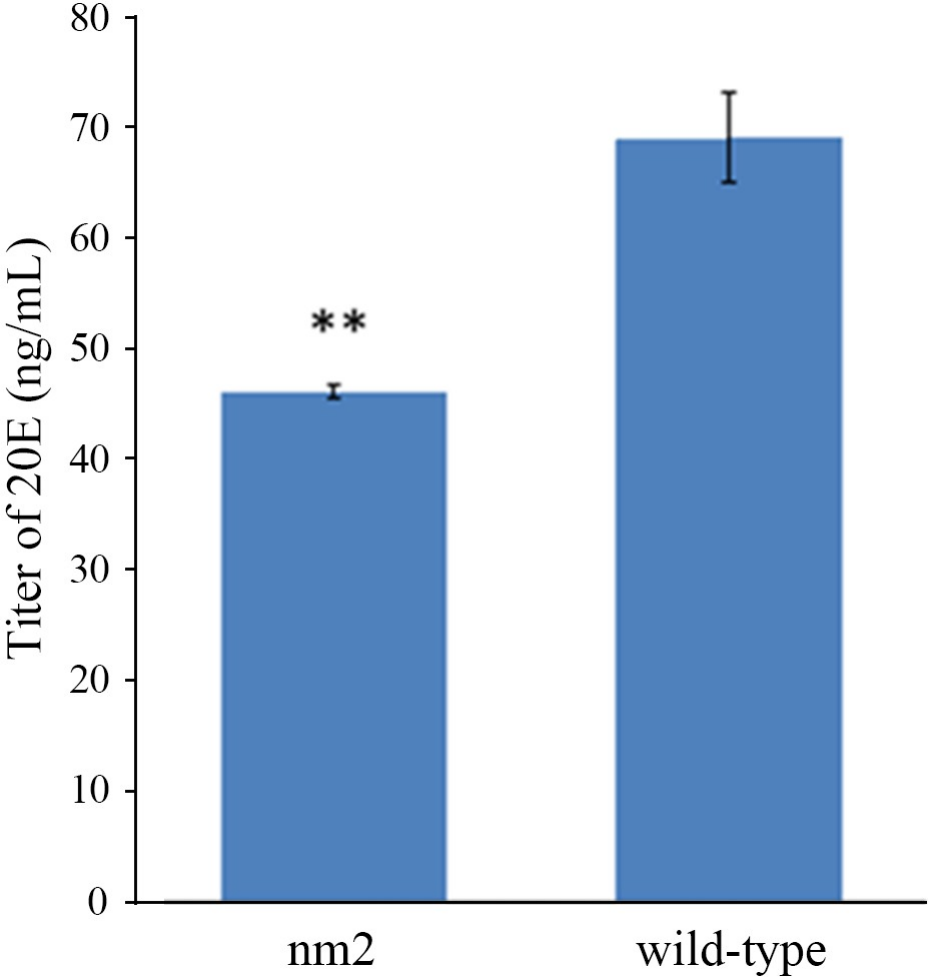
Assay of the ecdysone titre in the wild type and *nm2*, as determined by ELISA. ELISA analysis was repeated at least three times for each set of protein samples. Each point represents the mean value ±SD. **indicates significant difference (p < 0.01) compared with the wild type.

Based on the facts that the *BmCPG10* encoded a cuticle protein and that the expression of most cuticle protein genes were induced by an ecdysone pulse, the expression of the cuticle protein genes required the presence and elimination of 20E [20–25]. We speculated that the mutation of *BmCPG10* would have some influences on the ecdysone titre. Thus the titre of ecdysone in *nm2* and wild-type were determined by ELISA. The result indicated that ecdysone titre in *nm2* was significantly lower than that of the wild-type (Fig 5).

### The *nm2* mutant could be rescued by feeding 20E, 7dC and cholesterol

**Fig 6:**
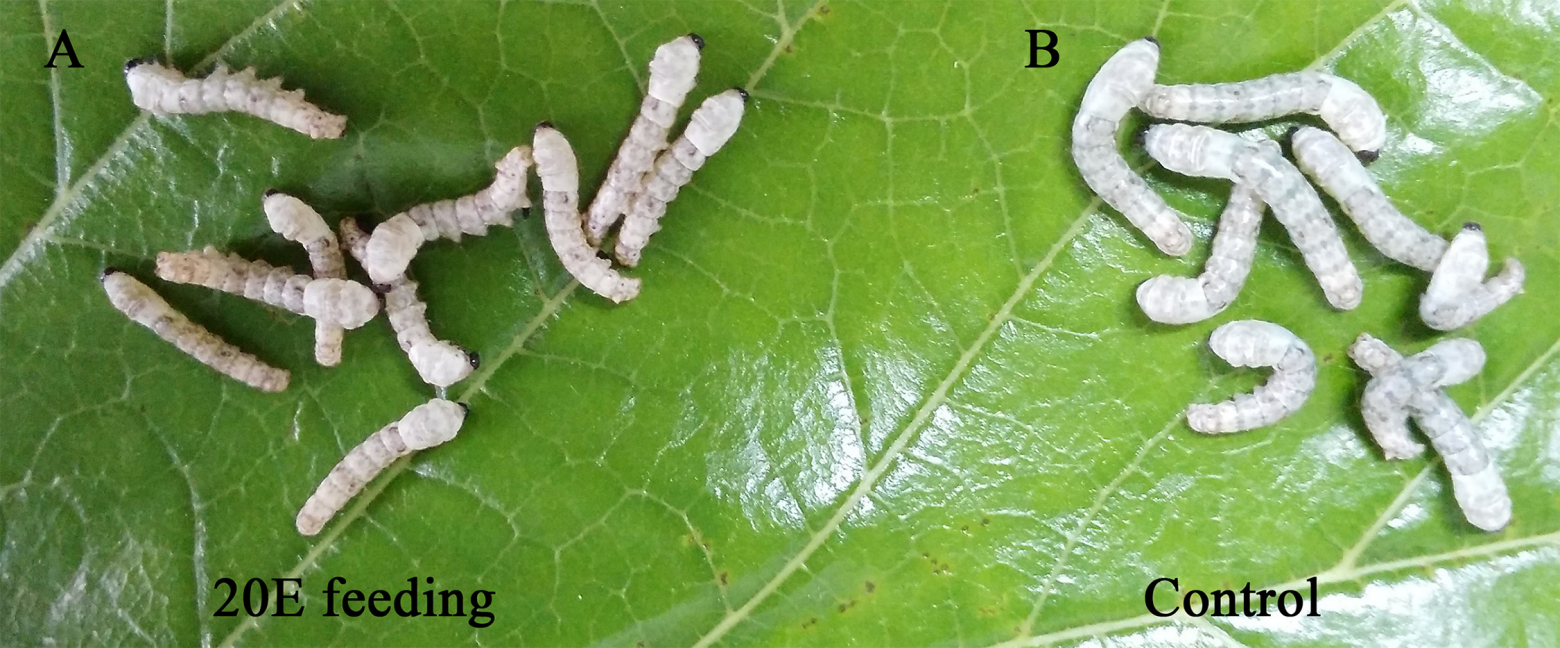
The feeding rescue experiments by 20E. Each feeding was repeated at least three times for each group of samples (N = 50). The left larvae were fed 20E and moulted in the 2^nd^ instar. The right larvae were the controls and could not moult in the 2^nd^ instar with a glossy cuticle phenotype.

Based on the difference of in the ecdysone titre between the wild-type and *nm2*, we speculated that the synthesis of ecdysone was affected by the mutation of *BmCPG10*. Cholesterol, 7dC and 20E, which are the most upstream materials, as well as the intermediate and end products for ecdysteroid biosynthesis, respectively, dissolved or suspended in water and were fed to each group, including 50 *nm2*; water was used as a control. As a result, 96% of the 50 *nm2* that were fed 8000 mg/L cholesterol, 40% of 50 *nm2* that were fed 8000 mg/L 7dC and 98% *nm2* that were fed 400 mg/L 20E moulted in the 2^nd^ instar and grew into the 3^rd^ instar stage but developed slowly in the third instar, with different sizes. These were then fed fresh mulberry leaves, and the partially rescued *nm2* then moulted and grew into the 4^th^ instar stage but could not moult in the 4^th^ instar and finally died (Fig 6). However, in controls that were given water, none of the all 50 *nm2* individuals could not moult in the 2^nd^ instar and subsequently died (Table 1). These results showed that the cholesterol that provided the raw materials for ecdysteroid biosynthesis was deficient in *nm2*, which was caused by the mutation of *BmCPG10*.

**Table 1:** Results of feeding rescue experiments with 20E, cholesterol, and 7dC, using water as a control.

Treatment	Total number of *nm2* larvae fed	Number to 2^nd^ moulting	Number to 3^rd^ moulting	Number to 4^th^ moulting
20E	50	49	23	0
Cholesterol	50	48	20	0
7dC	50	20	13	0
Water	50	0	-	-

## Discussion

In the present study, we fine mapped the candidate genes of the *nm2* mutant by positional cloning and identified the function to illustrate the *nm2* mutation at the molecular level. We focused on the two genes that had different mRNA expression between the wild type and *nm2*. One was *BMgn002601* and the other was *BmCPG10*. And the key gene responsible for the *nm2* was further determined by cloning of their ORF and the RNAi of *BmCPG10*. The ORF of *BMgn002601* was identical between wild type and *nm2*, but the *BmCPG10* ORF in *nm2* involved a deletion of 217 bp in its functional domain, in addition the mRNA expression of *BMgn002601* could be influenced by the expression of *BmCPG10*. Based on these results, we concluded that the mutation of *BmCPG10* was the most likely key gene responsible for the non-moulting in the 2^nd^ instar silkworm mutant.

There are various types of structural cuticle protein genes in insect genomes. Currently, in all of the sequenced insects, the proportion of cuticle protein genes in an insect’s total estimated protein-coding genes is more than 1% [19]. Insect cuticle, as a main component of exoskeleton [19, 26, 27], is very important for insect growth, reproduction, and adaptivity to the complex and changeable environment. The *BmCPG10* gene encodes a cuticle protein that is rich in glycine and belongs to the CPG family. This protein of the CPG family mainly exists in the hard stratum corneum. Similar to other structural proteins that are rich in glycine, such as chorionin, intermediate filaments, and cytokeratins, CPGs can provide protection and support for insects [28]. The repeat sequences of glycine, including GXGX、GGXG or GGGX, can form a soft curling structure [29], which is very important for the crosslinking of cuticle protein during hardening [30]. In the present study, the 217 bp deletion of *BmCPG10* was located in the functional domain that rich in glycine and include the repeat sequences of glycine, which might influence the crosslinking of cuticle protein during hardening. Okamoto et al. investigated the expression of 17 CPG genes during the last larval moult of *Bombyx mori*, and the result showed that all of the genes were abundantly expressed in the epidermis but were barely expressed in the fat body, haemocytes and midgut, suggesting that they were mainly involved in the construction of the newly synthesized cuticle of the epidermis [31]. The tissue expression of the *BmCPG10* gene in this study was similar to that of the Okamoto et al study, the *BmCPG10* gene transcription had high level in the epidermis, head and trachea, which suggested that the *BmCPG10* might participate in the synthesis and construction of these organs. However, *BmCPG10* mRNA was also expressed in midgut, Malpighian tubule, prothoracic gland and ventral nerve cord in the present study, which showed that *BmCPG10* had other functions in addition to synthesizing the cuticle of the epidermis.

The structure and composition of insect cuticle are renewed during moulting and metamorphosis, and the cuticle protein coding genes are regulated by Juvenile hormone and ecdysteroid because the cuticle proteins are the important part of the epidermis [32]. Many cuticle protein genes were reported to be up-regulated by 20E [33–37]. Okamoto studied the expression profiles of 17 cuticle protein genes at the 4^th^ moult; the results indicated that most of the cuticle protein genes tested were highly expressed when the ecdysone titre decreased or disappeared but were not expressed when the titre of ecdysone was high. Thus, the expression of cuticle protein genes was negatively correlated with the titre of ecdysone [31]. The expression of *BmCPG10* mRNA showed a similar tendency to the results of Okamoto. Because of the structural variation of *BmCPG10* in *nm2*, the mRNA expression and the encoded protein of *BmCPG10* were affected, and the titre of ecdysone was lower in *nm2* than that of in wild-type, which resulted in non-moulting. In the rescued experiment, the *nm2* mutant could be rescued by feeding 20E, cholesterol, 7dC; because cholesterol was slightly soluble in water, but 7dC was not dissolve in water and the different solubility might have some influence on the absorption and digestion of silkworm, so the rescued rate of 7dC was lower than that of the cholesterol. All of these results gave us a hint that the *BmCPG10* was closely related to the titre of ecdysone in silkworm.

Sora Enya reported a novel Halloween gene, *noppera-bo* (*nobo*), that encoded a member of the glutathione S–transferase family. The larvae that were knock-downs of the *nobo* gene displayed an arrested phenotype and a reduced 20E titre and could be rescued by the administration of 20E or cholesterol. The results presented the possibility that *nobo* played a crucial role in regulating the behaviour of cholesterol in the biosynthesis of steroids in insects [38]. The silkworm mutant nobo-Bm was non-moulting in the 2^nd^ instar and had a glossy cuticle phenotype, which could be rescued by the application of 20E, suggesting that the nobo family is essential for the regulation of sterol utilization in the Lepidoptera [13]. The silkworm mutant *nm2* in this study was very similar to nobo-Bm and could be rescued by feeding 20E and cholesterol, which might indicate that the mutation of *BmCPG10* had an influence on sterol utilization and resulted in acholesterol deficiency, which was the raw material required for the synthesis of ecdysone that were synthesized from dietary sterols. However, the specific molecular mechanism of *BmCPG10* warrants further research.

## Supporting Information

S1 Table Polymorphic SSR markers on each linkage group of silkworm. (DOC)

S2 Table Polymorphic SSR markers linked to *nm2* gene. (DOCX)

S3 Table The primers of the thirteen candidate genes for semi-quantitative RT-PCR. (DOCX)

S4 Table The preliminary result of feeding experiment with 20E, Cholesterol, and 7dC, using water as control. (DOCX)
