# Supplementary material for: Mutation of a Cuticle Protein Gene, BmCPG10, Is Responsible for Silkworm Non-Moulting in the 2nd Instar Mutant
Source: PLoS One. 2016 Apr 20;11(4):e0153549. doi: 10.1371/journal.pone.0153549 (PMC4838254; doi:10.1371/journal.pone.0153549)
Supplement: S1 Table — (DOC) [file pone.0153549.s001.doc]

Table S1 Polymorphic SSR markers on each linkage group of silkworm

| Marker No. | Location in the Silkworm | Primer sequences (5´-3´) |
| --- | --- | --- |
| Schr1-1690-6 | chr.1 | F:CAGCCGTTTAGGAGGACA  R: TGGCTTCGGTTACAGGTC |
| S0208 | Chr.2 | F: CCACGGCCAAGGCGAGA  R: CGTGGTGAGCAATACGTCCTG |
| Schr3-w-3 | Chr.3 | F: CAACATTCCATTTATGTCACCT  R: TATTCGTTGCTGCTGGGTCA |
| S0404 | Chr.4 | F: CGTTTGCCTACGCTTGCC  R: CACGCAGAGGCAGTTGTCG |
| S2529-2 | Chr.5 | F: CCCCTTCCAACACTACAT  R: ATCGTATCAAAGCGCACC |
| Schr6-140301-15 | Chr.6 | F:AAAGTACGTGTCGGATAG  R: CGAGAAAGTCAGTGGGTT |
| S0707 | Chr.7 | F: CGGAATCGTGACCCACTAAGAA  R: CCGGTTCATTTCAGAACTGTGG |
| S0810 | Chr.8 | F: CAGCGAAGCGGACGGGTA  R: GCCCCTCCATGCAATTTGTA |
| S0913 | Chr.9 | F: CATAATTGATGAGCAGACATGTATATAAAA  R: GGTGCGGCTGCTCCATTG |
| Schr10-w-4 | Chr.10 | F: CACTTTGATGTCGTGTTAAGG  R: CAGCACATGTATCTGACCTC |
| S1108 | Chr.11 | F: TTGAAGATAGAGCGAAGTGGAGG  R: GCGGCAGAAATAGGACGGT |
| Schr12-140301-5 | Chr.12 | F: GCTGCTACTTGCGGGATT  R: GATTCTTGACCACCTGATG |
| S1310 | Chr.13 | F: CAGTGCGAGTATTACAAGAAACAGATT  R: GCGATAAGACCGCCAATTGTA |
| S1403 | Chr.14 | F: TGAAGGACAAAAGGGAATGCC  R: TCGATTGTCGCCGGTTAACT |
| S1509 | Chr.15 | F: ACACGGACCATCTCTGGC  R: CCGAATTTCGGCGTTTTAA |
| S1603 | Chr.16 | F: CATGAGCACGCTCTTGGTTTC  R: ATCCGAAAATTAGAGCTGAGCAA |
| Schr17-w-4 | Chr.17 | F: AAAGCTATGACCTTCGCCCT  R: TGTTCCCGCAGAAATGTGATA |
| S1805 | Chr.18 | F: CGTGGAGCCGAAAGCGA  R: CGTATCTGCTTGTTGACTATTTTCTGA |
| S1911 | Chr.19 | F: GAATAACGCTAAAGGCGAATG  R: CAATGCCTTTCTTGGAATATGTATGA |
| Schr20-w-4 | Chr.20 | F: ACATGAACTTTGGAGAACAGAA  R: GGCATCATCAAGCCATTCAG |
| Schr21-140224-1 | Chr.21 | F: AAACGGTTGCCTGGAAGA  R:TCCCACTATTCCCACTCT |
| S2201 | Chr.22 | F: CACAATAAGTCCTACCAACAGTGAGAA  R: TGTTGAGCGACAGGAGGTTTTA |
| S2301 | Chr.23 | F:GCAGTACCAGCAGCTTGACG  R: CAAGGGTATCTCAAGCATAGACAGG |
| S2405 | Chr.24 | F: GGCAAGCCCAGAACAACGG  R: ACACGCTACATTTCACTTTGTTACG |
| S2505 | Chr.25 | F: GCGTCAACTACAAAGTCATACCCAA  R: ACGCATAAGCCCACAGCCT |
| S2603 | Chr.26 | F: CCGAAGCGACATATTTTGGG  R: CGTAGTTCGTTTCCAAATTCTCG |
| S2705 | Chr.27 | F: GTCGCATCTACCGCCTTCTAAT  R: AGCACAATGGACGAGTGGTTCT |
| S2853-7 | Chr.28 | F:CCAGTAGGCACGCAAAAGGAA  R: AATGCCAGGTTAAGAGTATGAAAGC |
