# Supplementary material for: Mutation of a Cuticle Protein Gene, BmCPG10, Is Responsible for Silkworm Non-Moulting in the 2nd Instar Mutant
Source: PLoS One. 2016 Apr 20;11(4):e0153549. doi: 10.1371/journal.pone.0153549 (PMC4838254; doi:10.1371/journal.pone.0153549)
Supplement: S2 Table — (DOCX) [file pone.0153549.s002.docx]

Table S2 Polymorphic SSR markers linked to *nm2* gene

| Marker No. | Location in the Silkworm | Primer sequences (5´-3´) | Exchange number /Total number of BC1M |
| --- | --- | --- | --- |
| S2529-35 | Nscaf2529:5796585-5796604 | F: TGTTAGCAACCCTGACCATT  R: CATTCTCCATCATCTATCCC | 44/594 |
| S2529-43 | Nscaf2529:5587896-5587915 | F: ATTGTACTGTGCCCGTCCTG  R: GGTGTTCCTCCCTCCCATTT | 36/594 |
| S2529-45 | Nscaf2529: 5526068 -5526088 | F: GATGTTCGGACCAAATAGCG  R: GGAGGTTGCGGTTAAAGGAG | 22/594 |
| S2529-46 | Nscaf2529:5520491-5520510 | F: GTCCCGTTTGGAGGTGTAAG  R: ACGGTGACTGTTCATGTGCC | 15/594 |
| S2529-32 | Nscaf2529:2772188-2772208 | F: AACTGACAGCGACATGAACA  R: AGCGATAAGACCGCCTATTT | 4/594 |
| S2529-27 | Nscaf2529:2496507-2496529 | F: GTGATCCTGCTACTGCTATACAC  R: CTCATTTCCTCAGTTTCGTC | 1/594 |
| S2529-22 | Nscaf2529:2211886-2211906 | F: CGACCACGGCTACACTACATC  R: TTGTATGTCGGTCCCTCCTC | 2/594 |
| S2529-91 | Nscaf2529:2154405-2154424 | F: AATAGTCCAGAGTAAAGTCC  R: TATTTGCGTAAAAGCCGTCT | 2/594 |
| S2529-2 | Nscaf2529:1267205-1267185 | F: CCCCTTCCAACACTACAT  R: ATCGTATCAAAGCGCACC | 8/594 |
| S2529-121 | Nscaf2529:1072879-1072898 | F: TAGCAGTCAGCAGGACGATA  R:ATAGCTTGCCTTTACTTTGG | 17/594 |
| S2529-74 | Nscaf2529:801855-801836 | F: TGTATCGCACTGTTTATTTG  R: GGCGCTTCATTTCAACATTT | 25/594 |
| S2529-104 | Nscaf2529:712926-712909 | F: GATGTCTATGTGCTCCAGTA  R: CAGAATACGCAACGATCAAG | 36/594 |
