# Supplementary material for: Mutation of a Cuticle Protein Gene, BmCPG10, Is Responsible for Silkworm Non-Moulting in the 2nd Instar Mutant
Source: PLoS One. 2016 Apr 20;11(4):e0153549. doi: 10.1371/journal.pone.0153549 (PMC4838254; doi:10.1371/journal.pone.0153549)
Supplement: S3 Table — (DOCX) [file pone.0153549.s003.docx]

Table S3 The primers of the thirteen candidate genes for semi-quantitative RT-PCR

| gene | Primer sequences(5´-3´) | gene | Primer sequences(5´-3´) |
| --- | --- | --- | --- |
| *BMgn002598* | F: TAGCGGCGAAGAAACTTCAGCTGTC  R: CGTATGTTACGATTGACGAGCGTCG | *BMgn002601* | F: TTCCGACCGAACAAGAAGCCTTGGA  R: GGTATATCTCTCAGTCTATGGCGGC |
| *BMgn002690* | F: GTAACAGTGCTCATCGTGGTTTCCG  R: GTCTTCTGTCCGTTGATGTCTGACG | *BmCPG10* | F: CTAGAACTCATGTAACGGACCGAGC  R: CGTATCCGTGGTGGCCTTTTCTGTA |
| *BMgn015078* | F: TGTCATTAATCACCATCGCGAGCGC  R: GCAACCATAGTTCCCTTCCCTCCAT | *BMgn002603* | F: GTTTGCTGTATCGTCGTTGAGGCAC  R: AGAGCGGCTATCACCAATTTCTGGG |
| *BMgn002599* | F: AACAACATTCGGACCGTCGGTCTAC  R: GCTTTTATCTGCGAGAATGCGGGGA | *BMgn002687* | F: AAGGGCACCAGTTCCCCAGCAATTT  R: ATGTGCGTGTATTCCATGCCCTGAC |
| *BMgn002689* | F: ATTACAATCGCGAGCGCTGGCTTTG  R: CTGTAACTCGACTTAGACTCTCCGG | *BMgn0015079* | CGGCACTGTCATGGCATAGTATTCG  TTGGGCGTTCCAGTCTAACACGTAT |
| *BMgn002600* | F: CACCTCCCGTCTTGCCTGTTTTAGA  R:AGAACGACTGAGAAGCGGAGTGACT | *BMgn002685* | F:GCCAGAAAGATACCACCTGGACCAA  R:TAGCGAGGTTAACGTTGGCCTTCCT |
| *BMgn002688* | F:AGCCGGTTGAAACTAACAACGGAGG  R: TTGGTGTGTTTCACGAGTGCTTGGC |  |  |
