# Supplementary material for: Mutation of a Cuticle Protein Gene, BmCPG10, Is Responsible for Silkworm Non-Moulting in the 2nd Instar Mutant
Source: PLoS One. 2016 Apr 20;11(4):e0153549. doi: 10.1371/journal.pone.0153549 (PMC4838254; doi:10.1371/journal.pone.0153549)
Supplement: S4 Table — (DOCX) [file pone.0153549.s004.docx]

Table S4 The preliminary result of feeding experiment with 20E, Cholesterol, and 7dC, using water as control

| Treatment | Concentration  (mg/L) | Total number of nm2 larvae fed | Number to undergo 2^nd^ moulting | Number to undergo 3^rd^ moulting | Number to undergo 4^th^ moulting |
| --- | --- | --- | --- | --- | --- |
| 20E | 100 | 20 | 3 | 1 | 0 |
|  | 200 | 20 | 5 | 2 | 0 |
|  | 400 | 20 | 18 | 11 | 0 |
| Cholesterol | 2000 | 20 | 5 | 1 | 0 |
|  | 4000 | 20 | 8 | 3 | 0 |
|  | 8000 | 20 | 17 | 9 | 0 |
| 7dC | 2000 | 20 | 1 | 0 | 0 |
|  | 4000 | 20 | 3 | 1 | 0 |
|  | 8000 | 20 | 8 | 5 | 0 |
| Water |  | 20 | 0 | 0 | 0 |
